# The clinical, molecular, and therapeutic implications of time from primary diagnosis to brain metastasis in lung and breast cancer patients

**DOI:** 10.1002/cam4.7364

**Published:** 2024-06-07

**Authors:** Haitao Ge, Kaibin Zhu, Qian Sun, Huan Wang, Hui Liu, Jinyi Ge, Chunyang Liu, Peng Liang, Zhonghua Lv, Hongbo Bao

**Affiliations:** ^1^ Department of Neurosurgery The First Affiliated Hospital of Harbin Medical University Harbin China; ^2^ Department of Thoracic Surgery Harbin Medical University Cancer Hospital Harbin China; ^3^ Department of Neurosurgery Harbin Medical University Cancer Hospital Harbin China; ^4^ Harbin Medical University Harbin China; ^5^ Department of Neurosurgery, Beijing Tiantan Hospital Capital Medical University Beijing China

**Keywords:** brain metastasis, breast cancer, interval time, lung cancer, primary diagnosis

## Abstract

**Purpose:**

Lung cancer (LC) and breast cancer (BC) are the most common causes of brain metastases (BMs). Time from primary diagnosis to BM (TPDBM) refers to the time interval between initial LC or BC diagnosis and development of BM. This research aims to identify clinical, molecular, and therapeutic risk factors associated with shorter TPDBM.

**Methods:**

We retrospectively reviewed all diagnosed LC and BC patients with BM at Harbin Medical University Cancer Hospital from 2016 to 2020. A total of 570 patients with LC brain metastasis (LCBM) and 173 patients with breast cancer brain metastasis (BCBM) patients who met the inclusion criteria were enrolled for further analysis. BM free survival time curves were generated using Kaplan–Meier analyses. Univariate and multivariate Cox regression analyses were applied to identify risk factors associated with earlier development of BM in LC and BC, respectively.

**Results:**

The median TPDBM was 5.3 months in LC and 44.4 months in BC. In multivariate analysis, clinical stage IV and M1 stage were independent risk factors for early development of LCBM. LC patients who received chemotherapy, targeted therapy, pulmonary radiotherapy, and pulmonary surgery had longer TPDBM. For BC patients, age ≥ 50 years, Ki67 ≥ 0.3, HER2 positive or triple‐negative breast cancer subtype, advanced N stage, and no mastectomy were correlated with shorter TPDBM.

**Conclusions:**

This single‐institutional study helps identify patients who have a high risk of developing BM early. For these patients, early detection and intervention could have clinical benefits.

## Graphical abstract

This study investigates the interval between the primary diagnosis and the development of brain metastases in lung and breast cancer patients, highlighting risk factors associated with shorter intervals. Employing retrospective analysis of patient data, the study identifies clinical, molecular, and therapeutic predictors that could inform early intervention strategies to potentially enhance clinical outcomes in high‐risk patients.

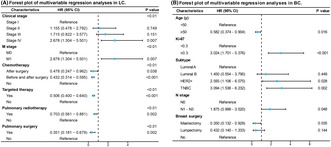

## 1 INTRODUCTION

Lung cancer (LC) and breast cancer (BC) have been the most prevalent types of cancer worldwide for decades.^1^ LC and BC are also the most common primary tumors that metastasize to the brain, accounting for an average of 45% and 15% of all brain metastases (BMs), respectively.^2^ In the vast majority of cases, the appearance of BM is a sign of poor prognosis. For instance, only one in four LCBM patients survives beyond 1 year after diagnosis.^3^ For BCBM, the 1‐year survival rate is approximately 20%.^4^ Despite advances in targeted therapy and immunotherapy, only a limited number of BM patients benefit from these novel treatment methods.^5^ Moreover, improvements in primary tumor control and an aging population contribute to the elevated incidence of BM.^6^ Therefore, early detection and diagnosis are important in BM management.

Several previous studies focused on exploring risk indicators for BM. As they demonstrated, molecular features are one of the critical factors that affect BM incidence. LC patients with epidermal growth factor receptor (EGFR) mutation^7^ and BC patients whose human epidermal growth factor receptor 2 (HER2) is positive^8^ tend to develop BM more frequently. Some clinical features, such as the diameter of the primary lung lesion and carcinoembryonic antigen (CEA) concentration, could also serve as independent risk factors for LCBM.^9^ In addition to the abovementioned factors, the timing of BM, which varies widely in clinical work, deserves attention. In some cases, metastatic tumors are found synchronously with or even before primary cancer.^10^ This early dissemination is more prevalent in LC and melanoma.^11^ In contrast, disseminated cancer cells may live in the brain microenvironment for years in a latent state until they are activated, finally leading to the development of BM.^12^, ^13^ A few studies have investigated the risk factors associated with a shorter time from primary diagnosis to BM (TPDBM) in LC and BC patients, but they mainly focus on tumor pathological subtypes.^14^, ^15^, ^16^ Early diagnosis and treatment of BM could reduce complications and prolong metastatic survival.^17^ Hence, it is vital to screen the factors involved in the development of BM. In clinical work, a patient with shorter TPDBM equates to the patient developing BM in the early stage of the primary lesion, and these patients in particular require special attention. A better understanding of the factors that impact TPDBM in LC and BC may contribute to clinical practice and early intervention with treatment.

The purpose of this study was to describe how clinical features, molecular alterations, and therapy‐related factors affect TPDBM in patients with LC and BC with the hope of identifying patients at high‐risk of shorter TPDBM who would benefit from early screening and intervention. Compared with previous studies, our research covered all BC and LC subtypes and collected detailed information on treatment regimens that were in accordance with current standard management, making this work more instructive for clinical decision‐making.

## 2 METHODS

### 2.1 Patients

This research was ethically approved by the Ethics Committee of Harbin Medical University Cancer Hospital (#KY2021‐42) and conducted by the Declaration of Helsinki. We retrospectively reviewed and analyzed all patients who were diagnosed with subsequent BM from LC or BC between 2016 and 2020 at our hospital. During this period, 27,617 patients with LC and 10,449 patients with BC received treatment at our center. Among them, 3202 LC patients and 733 BC patients were also diagnosed with BM. Patients with BM enrolled in this study met the following criteria^1^: original histologic or molecular reports were available to confirm the diagnosis of LC or BC^2^; pathological diagnosis or radiographic evidence of BM^3^; generally complete clinical data; and^4^ no previous history of other malignancies. Finally, 570 patients with LCBM (Table 1) and 173 patients with BCBM (Table 2) were screened for eligibility. Written informed consent was obtained from all patients.

**TABLE 1:** Main characteristics of LCBM patients.

	Total	LUAD	LUSC	SCLC	Others	*p* value
Patients with LC						
Patients, *n* (%)	570 (100%)	343 (60.2)	43 (7.5)	175 (30.7)	9 (1.6)	–
Median age, year (mean ± SD)	57.86 ± 9.13	57.48 ± 9.37	58.63 ± 6.73	58.54 ± 9.06	55.89 ± 11.16	0.514
Median TPDBM, month (range)	5.3 (0.6–13.5)	3.0 (0.5–16.5)	5.5 (2.0–13.6)	7.5 (3.0–11.3)	5.0 (3.2–25.3)	0.044
Gender						
Male	312 (54.7)	149 (43.4)	33 (76.7)	124 (70.9)	6 (66.7)	<0.001
Female	258 (45.3)	194 (56.6)	10 (23.3)	51 (29.1)	3 (33.3)	
Clinical stage, *n* (%)						
Stage I	12 (2.1)	6 (1.7)	1 (2.3)	5 (2.9)	0 (0.0)	0.005
Stage II	15 (2.6)	8 (2.3)	2 (4.7)	4 (2.3)	1 (11.1)	
Stage III	82 (14.4)	38 (11.1)	10 (23.3)	32 (18.3)	2 (22.2)	
Stage IV	384 (67.4)	264 (77.0)	26 (60.5)	90 (51.4)	4 (44.4)	
Unknown	77 (13.5)	27 (7.9)	4 (9.3)	44 (25.1)	2 (22.2)	
T stage, *n* (%)						
T1	181 (31.8)	126 (36.7)	16 (37.2)	37 (21.1)	2 (22.2)	0.185
T2	131 (23.0)	93 (27.1)	8 (18.6)	28 (16.0)	2 (22.2)	
T3	40 (7.0)	21 (6.1)	8 (18.6)	11 (6.3)	0 (0.0)	
T4	46 (8.1)	28 (8.2)	4 (9.3)	12 (6.9)	2 (22.2)	
Unknown	172 (30.2)	75 (21.9)	7 (16.3)	87 (49.7)	3 (33.3)	
N stage, *n* (%)						
N0	74 (13.0)	49 (14.3)	6 (14.0)	19 (10.9)	0 (0.0)	0.010
N1	23 (4.0)	15 (4.4)	4 (9.3)	3 (1.7)	1 (11.1)	
N2	210 (36.8)	123 (35.9)	21 (48.8)	61 (34.9)	5 (55.6)	
N3	106 (18.6)	84 (24.5)	5 (11.6)	17 (9.7)	0 (0.0)	
Unknown	157 (27.5)	72 (21.0)	7 (16.3)	75 (42.9)	3 (33.3)	
M stage, *n* (%)						
M0	111 (19.5)	52 (15.2)	13 (30.2)	43 (24.6)	3 (33.3)	<0.001
M1	305 (53.5)	222 (64.7)	23 (53.5)	57 (32.6)	3 (33.3)	
Unknown/uncertain	154 (27.0)	69 (20.1)	7 (16.3)	75 (42.9)	3 (33.3)	
Chemotherapy, *n* (%)						
After surgery	46 (8.1)	32 (9.3)	9 (20.9)	4 (2.3)	1 (11.1)	<0.001
Before and after surgery	437 (76.7)	236 (68.6)	30 (69.8)	165 (94.3)	6 (66.7)	
No	84 (14.7)	72 (21.0)	4 (9.3)	6 (3.4)	2 (22.2)	
Unknown	3 (0.5)	3 (0.9)	0 (0.0)	0 (0.0)	0 (0.0)	
Targeted therapy, *n* (%)						
Yes	174 (30.5)	171 (49.8)	2 (4.7)	0 (0.0)	1 (11.1)	<0.001
No	392 (68.8)	168 (49.0)	41 (95.3)	175 (100.0)	8 (88.9)	
Unknown	4 (0.7)	4 (1.2)	0 (0.0)	0 (0.0)	0 (0.0)	
Pulmonary radiotherapy, *n* (%)						
Yes	221 (38.8)	99 (28.9)	18 (41.9)	103 (58.9)	1 (11.1)	<0.001
No	349 (61.2)	244 (71.1)	25 (58.1)	72 (41.1)	8 (88.9)	
Pulmonary surgery, *n* (%)						
Yes	63 (11.1)	44 (12.8)	11 (25.6)	6 (3.4)	2 (22.2)	<0.001
No	507 (88.9)	299 (87.2)	32 (74.4)	169 (96.6)	7 (77.8)	
EGFR status, *n* (%)						
Wild type	80 (23.3)	80 (23.3)	–	–	–	–
Mutation	131 (38.2)	131 (38.2)	–	–	–	–
Unknown	132 (38.5)	132 (38.5)	–	–	–	–

**TABLE 2:** Main characteristics of BCBM patients.

	Total	Luminal A	Luminal B	HER2+	TNBC	*p* value
Patients with BCBM						
Patients, *n* (%)	173 (100)	61 (35.3)	21 (12.1)	60 (34.7)	31 (17.9)	–
Median age, years (mean ± SD)	52.03 ± 11.11	51.69 ± 11.20	54.19 ± 11.49	51.48 ± 10.10	52.29 ± 11.30	0.801
Median TPDBM, month (range)	44 (28–77.5)	56 (36–99.5)	68 (36–102.5)	39 (23–62)	33.5 (18.3–56.3)	0.002
ER status, *n* (%)						
Positive	108 (62.4)	61 (100.0)	15 (71.4)	32 (53.3)	0 (0.0)	<0.001
Negative	65 (37.6)	0 (0.0)	6 (28.6)	28 (46.7)	31 (100.0)	
PR status, *n* (%)						
Positive	92 (53.2)	61 (100.0)	6 (28.6)	25 (41.7)	0 (0.0)	<0.001
Negative	81 (46.8)	0 (0.0)	15 (71.4)	35 (58.3)	31 (100.0)	
HER2 status, *n* (%)						
Positive	60 (34.7)	0 (0.0)	0 (0.0)	60 (100.0)	0 (0.0)	<0.001
Negative	113 (65.3)	61 (100.0)	21 (100.0)	0 (0.0)	31 (100.0)	
Ki‐67 (range)	0.3 (0.1–0.5)	0.15 (0.05–0.3)	0.25 (0.05–0.6)	0.3 (0.11–0.44)	0.5 (0.25–0.8)	0.001
Clinical stage, *n* (%)						
Stage I	10 (5.8)	3 (4.9)	1 (4.8)	3 (5.0)	3 (9.7)	0.288
Stage II	71 (41.0)	28 (45.9)	6 (28.6)	20 (33.3)	17 (54.8)	
Stage III	24 (13.9)	6 (9.8)	3 (14.3)	13 (21.7)	2 (6.5)	
Stage IV	1 (0.6)	0 (0.0)	0 (0.0)	1 (1.7)	0 (0.0)	
Unknown	67 (38.7)	24 (39.3)	11 (52.4)	23 (38.3)	9 (29.0)	
T stage, *n* (%)						
T1	25 (14.5)	10 (16.4)	2 (9.5)	8 (13.3)	5 (16.1)	0.551
T2	65 (37.6)	25 (41.0)	5 (23.8)	21 (35.0)	14 (45.2)	
T3	10 (5.8)	1 (1.6)	1 (4.8)	5 (8.3)	3 (9.7)	
T4	4 (2.3)	1 (1.6)	1 (4.8)	2 (3.3)	0 (0.0)	
Unknown	69 (39.9)	24 (39.3)	12 (57.1)	24 (40.0)	9 (29.0)	
N stage, *n* (%)						
N0	28 (16.2)	8 (13.1)	2 (9.5)	9 (15.0)	9 (29.0)	0.698
N1	60 (34.7)	23 (37.7)	7 (33.3)	18 (30.0)	12 (38.7)	
N2	12 (6.9)	4 (6.6)	1 (4.8)	6 (10.0)	1 (3.3)	
N3	7 (4.0)	2 (3.3)	1 (4.8)	4 (6.7)	0 (0.0)	
Unknown	66 (38.2)	24 (39.3)	10 (47.6)	23 (38.3)	9 (29.0)	
M stage, *n* (%)						
M0	105 (60.7)	37 (60.7)	10 (47.6)	36 (60.0)	22 (71.0)	0.409
M1	2 (1.2)	0 (0.0)	1 (4.8)	1 (1.7)	0 (0.0)	
Unknown	66 (38.2)	24 (39.3)	10 (47.6)	23 (38.3)	9 (29.0)	
Chemotherapy, *n* (%)						
Yes	150 (86.7)	52 (85.2)	20 (95.2)	49 (81.7)	29 (93.5)	0.416
No	22 (12.7)	8 (13.1)	1 (4.8)	11 (18.3)	2 (6.5)	
Unknown	1 (0.6)	1 (1.6)	0 (0.0)	0 (0.0)	0 (0.0)	
Neoadjuvant chemotherapy, *n* (%)						
Yes	35 (20.2)	8 (13.1)	3 (14.3)	17 (28.3)	7 (22.6)	0.178
No	138 (79.8)	53 (86.9)	18 (85.7)	43 (71.7)	24 (77.4)	
Unknown	0 (0.0)	0 (0.0)	0 (0.0)	0 (0.0)	0 (0.0)	
Endocrine therapy, *n* (%)						
Yes	63 (36.4)	35 (57.4)	10 (47.6)	14 (23.3)	4 (12.9)	<0.001
No	108 (62.4)	24 (39.3)	11 (52.4)	46 (76.7)	27 (87.1)	
Unknown	2 (1.1)	2 (3.3)	0 (0.0)	0 (0.0)	0 (0.0)	
Targeted therapy, *n* (%)						
Yes	50 (28.9)	4 (6.6)	0 (0.0)	45 (75.0)	1 (3.2)	<0.001
No	121 (69.9)	55 (90.2)	21 (100.0)	15 (25.0)	30 (96.8)	
Unknown	2 (1.2)	2 (3.3)	0 (0.0)	0 (0.0)	0 (0.0)	
Breast radiotherapy, *n* (%)						
Yes	97 (56.1)	34 (55.7)	13 (61.9)	33 (55.0)	17 (54.8)	0.886
No	74 (42.8)	25 (41.0)	8 (38.1)	27 (45.0)	14 (45.2)	
Unknown	2 (1.2)	2 (3.3)	0 (0.0)	0 (0.0)	0 (0.0)	
Breast surgery, *n* (%)						
Mastectomy	131 (75.7)	45 (73.8)	18 (85.7)	43 (71.7)	25 (80.6)	0.455
Lumpectomy	20 (11.6)	10 (16.4)	2 (9.5)	5 (8.3)	3 (9.7)	
No	22 (12.7)	6 (9.8)	1 (4.8)	12 (20.0)	3 (9.7)	

### 2.2 LC and BC subtypes

According to the 2021 WHO classification of lung tumors,^18^ the pathologic classification of LC includes small cell lung cancer (SCLC, *n* = 175) and non‐small‐cell lung cancer (NSCLC, *n* = 395). In all NSCLC cases, 343 were diagnosed as lung adenocarcinoma (LUAD), 43 lung squamous cell carcinoma (LUSC), and 5 as pulmonary large‐cell neuroendocrine carcinomas (LCNEC), and 4 as adenosquamous carcinomas. According to estrogen receptor (ER), progesterone receptor (PR), and HER2 status, BC can be divided into four subtypes: luminal A (ER+/PR+/HER2+) (*n* = 61), luminal B (ER+/PR−/HER2+) (*n* = 21), HER2+ (ER−/PR−/HER2+) (*n* = 60), and triple‐negative breast cancer (TNBC) (ER−/PR−/HER2−) (*n* = 31).^19^, ^20^

### 2.3 Tumor‐node‐metastasis classification

The Tumor‐node‐metastasis (TNM) classification was performed according to the standard criteria of the 8th TNM staging system.^21^ T stage refers to invasion into adjacent tissue; N stage refers to regional lymph node involvement, and M stage refers to distant metastases of the primary tumor.

### 2.4 Statistical analysis

TPDBM was defined as the interval time in months from diagnosis of primary LC or BC until the diagnosis of BM. If primary cancer and BM were diagnosed simultaneously, TPDBN was recorded as a value of 0. BM‐free survival time according to different pathologic types was estimated by the Kaplan–Meier curve and compared by a log‐rank test using R software (version 3.6.3). Descriptive statistics were performed to summarize the baseline characteristics of the patients. Independent sample *t*‐tests were performed for continuous variables. Categorical variables were analyzed using chi‐Square or Fisher exact tests. Univariate and multivariable Cox regression analyses were conducted to assess risk factors for shorter TPDBM. The following variables were investigated in the hazards model: age, sex, pathologic subtype, molecular alterations, clinical stage, TNM classification at initial diagnosis of the primary tumor, and subsequent therapies. Variables with *p* < 0.10 in the univariate analyses were entered into the multivariate analysis. The hazard ratios (HRs) with 95% confidence intervals (CI) estimated from the univariate and multivariate Cox regression analyses were used to identify longer (HR <1) and shorter (HR >1) TPDBM. A *p value* <0.05 was considered significant. These data were analyzed using SPSS software (version 22.0; SPSS Inc., Chicago, IL, USA). For significant results in the multivariate Cox regression analyses, we visualized the results using forest plots drawn with the ggplot2 package (version 3.3.6) in R software (version 4.2.1).

## 3 RESULTS

### 3.1 Patient characteristics

A total of 570 patients with LCBM and 173 patients with BCBM from Harbin Medical University Cancer Hospital (Harbin, China) were identified. Their baseline characteristics are summarized in Tables 1 and 2. There were 66 patients with a concurrent diagnosis of LC and BM in this study. In LCBM cases, LUAD occupied the largest proportion of the cohort (60.2%), followed by SCLC (30.7%), LUSC (7.5%), and other pathologic subtypes (1.6%). Patients showed significant differences in sex, clinical stage, TNM classification, and therapeutic method distribution. For BCBM patients, luminal A, luminal B, HER2+ and TNBC accounted for 35.3%, 12.1%, 34.7%, and 17.9%, respectively. Significant differences in molecular status (*p* < 0.001), Ki‐67 expression (*p* = 0.001), endocrine therapy (*p* < 0.001), and target therapy (*p* < 0.001) were observed.

### 3.2 Interval time to BM

**FIGURE 1:**
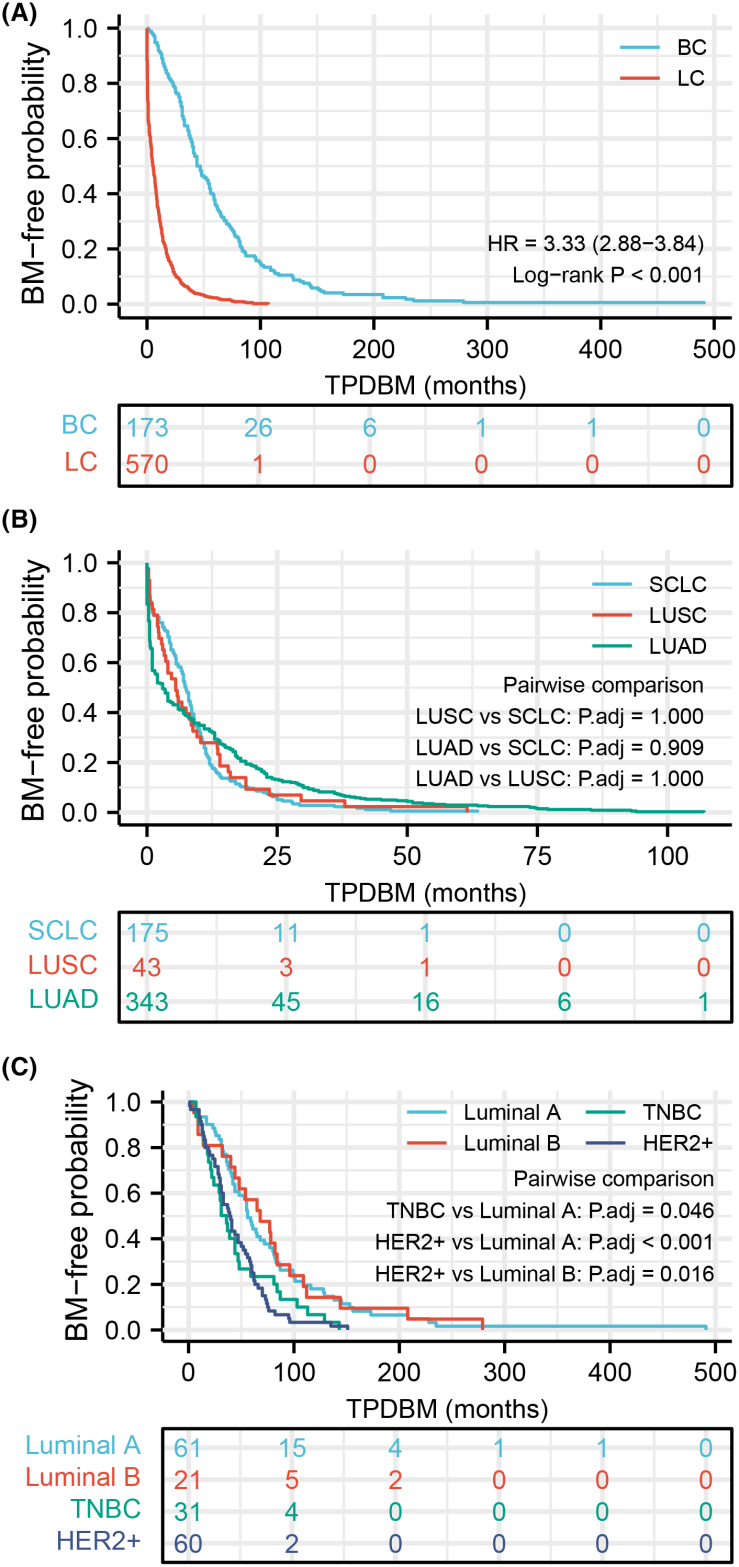
Kaplan–Meier estimates for BM‐free survival. BM‐free survival curve for (A) LC and BC, (B) LC subtypes, and (C) BC subtypes. BC, breast cancer; BM, brain metastasis; HER2, human epidermal growth factor receptor 2; LC, lung cancer; LUAD, lung adenocarcinoma; LUSC, lung squamous cell carcinoma; NSCLC, non‐small‐cell lung cancer; TNBC, triple‐negative breast cancer; TPDBM, time from primary diagnosis to BM.

As shown in Figure 1, BC patients had a median TPDBM of 44 months (interquartile range [IQR]: 28–77.5), which was much longer than that of LC patients (median 5.3 months, IQR: 4.0–6.8, *p* < 0.001). The median TPDBM was 7.5 months (IQR: 3.0–11.3) in SCLC and 3.5 months (IQR: 0.5–14.6) in NSCLC. Among all NSCLC subtypes, the median time to develop BM in LUSC was the longest (5.5 months, IQR: 2.0–13.6), whereas LUAD was the shortest (3.0 months, IQR: 0.5–16.5). However, there was no statistical difference in BM‐free survival curves between LC subgroups (Figure 1B). Similar to previous studies, we also found that TPDBM differed according to primary BC subtypes. The median TPDBM was 56 (IQR: 33–99.5) months in luminal A, 68 (IQR: 36–102.5) months in luminal B, 39 (IQR: 23–62) months in HER2+, and 33.5 (IQR: 18.3–56.3) months in TNBC. As indicated by Figure 1C, a significant difference was observed among the four subgroups (TNBC vs. luminal A, *p* = 0.046; HER2+ vs. luminal A, *p* < 0.001; HER2+ vs. luminal B, *p* = 0.016).

### 3.3 Univariate and multivariate analysis in LC

**FIGURE 2:**
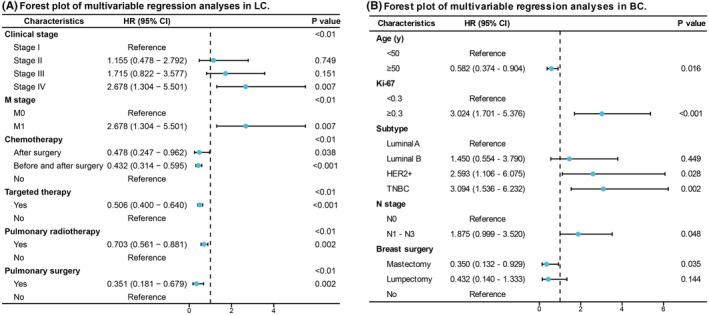
Forest plot of regression analyses. Forest plot of multivariable regression analyses in LC (A) and BC (B). CI, confidence interval; HR, hazard ratio.

Significant risk indicators of shorter TPDBM in all LC patients were screened from the univariate analysis: clinical stage, T2 stage, advanced N stage, M1 stage, chemotherapy, target therapy, pulmonary radiotherapy, and pulmonary surgery (Table 3). In multivariate analysis, clinical stage IV (HR = 2.678, *p* = 0.007) and M1 stage (HR = 2.678, *p* = 0.007) at initial diagnosis remained independent risk factors for shorter TPDBM. In contrast, our results indicated that patients who received chemotherapy after surgery (HR = 0.478, *p* = 0.038), chemotherapy prior to and after surgery (HR = 0.432, *p* < 0.001), targeted therapy (HR = 0.506, *p* < 0.001), pulmonary radiotherapy (HR = 0.703, *p* = 0.002), and pulmonary surgery (HR = 0.351, *p* = 0.002) developed BM later. Age, sex, pathologic subtype, and T stage were not statistically associated with TPDBM in the final results. The final significant results are shown in the forest plot in Figure 2A.

**TABLE 3:** Univariate and multivariate analysis of TPDBM in LC.

	Univariate analysis			Multivariate analysis		
	HR	95% CI	*p* value	HR	95% CI	*p* value
Age (years)						
<60	Reference	–	–	–	–	–
≥60	1.023	0.865–1.209	0.794	–	–	–
Gender						
Female	Reference	–	–	–	–	–
Male	1.091	0.924–1.287	0.304	–	–	–
Subtype						
LUAD	Reference	–	–	–	–	–
LUSC	1.076	0.782–1.480	0.653	–	–	–
SCLC	1.091	0.905–1.315	0.359	–	–	–
Others	0.698	0.359–1.357	0.289	–	–	–
Clinical stage						
Stage I	Reference	–	–	Reference	–	–
Stage II	1.151	0.537–2.465	0.718	1.155	0.478–2.792	0.749
Stage III	1.550	0.844–2.847	0.157	1.715	0.822–3.577	0.151
Stage IV	2.994	1.680–5.336	<0.001	2.678	1.304–5.501	0.007
T stage						
T1	Reference	–	–	Reference	–	–
T2	1.292	1.030–1.620	0.026	1.169	0.925–1.479	0.192
T3	1.042	0.736–1.475	0.817	1.249	0.866–1.803	0.235
T4	0.994	0.719–1.375	0.973	0.901	0.646–1.258	0.541
N stage						
N0	Reference	–	–	Reference	–	–
N1–N3	1.3	1.008–1.675	0.043	1.281	0.958–1.714	0.095
M stage						
M0	Reference	–	–	Reference	–	–
M1	2.197	1.757–2.747	<0.001	2.678	1.304–5.501	0.007
Chemotherapy						
After surgery	0.250	0.171–0.366	<0.001	0.478	0.247–0.962	0.038
Before and after surgery	0.685	0.542–0.867	0.002	0.432	0.314–0.595	<0.001
No	Reference	–	–	Reference	–	–
Targeted therapy						
Yes	0.697	0.580–0.837	<0.001	0.506	0.400–0.640	<0.001
No	Reference	–	–	Reference	–	–
Pulmonary radiotherapy						
Yes	0.812	0.685–0.963	0.017	0.703	0.561–0.881	0.002
No	Reference	–	–	Reference	–	–
Pulmonary surgery						
Yes	0.354	0.267–0.469	<0.001	0.351	0.181–0.679	0.002
No	Reference	–	–	Reference	–	–

For LUAD patients, we were curious about whether EGFR mutation alterations would affect TPDBM. Although EGFR mutation was associated with TPDBM in univariate analysis (HR = 0.617, *p* = 0.001), no significant results were found in multivariate analysis (HR = 0.811, *p* = 0.309, see Table S1 for details). In addition, clinical stage, metastasis, and surgery were no longer predictive indicators for TPDBM in LUAD.

### 3.4 Univariate and multivariate analysis in BC

We subsequently investigated underlying factors that might influence the development of BM in BC. In univariate analysis (Table 4), patients with younger age, higher expression of the Ki67, HER2+ or TNBC subtype, advanced clinical stage, more lymph node involvement, and no endocrine therapy had a high risk of shorter TPDBM. The final multivariate analysis results demonstrated that age ≥ 50 years (HR = 0.582, *p* = 0.016), Ki‐67 ≥ 0.3 (HR = 3.024, *p* < 0.001), HER2+ subtype (HR = 2.593, *p* = 0.0028), TNBC subtype (HR = 3.094, *p* = 0.002), N1–N3 stage (HR = 1.875, *p* = 0.048), and mastectomy (HR = 0.350, *p* = 0.035) were significantly associated with time to BCBM (Table 4; Figure 2B). Whether patients received chemotherapy, neoadjuvant chemotherapy, endocrine therapy, and targeted therapy or not had no obvious impact on TPDBM.

**TABLE 4:** Univariate and multivariate analysis of TPDBM in BC.

	Univariate analysis			Multivariate analysis		
	HR	95% CI	*p* value	HR	95% CI	*p* value
Age (years)						
<50	Reference	–	–	Reference	–	–
≥50	0.717	0.523–0.983	0.039	0.582	0.374–0.904	0.016
Ki‐67						
<0.3	Reference	–	–	Reference	–	–
≥0.3	2.145	1.549–2.972	<0.001	3.024	1.701–5.376	<0.001
Subtype						
Luminal A	Reference	–	–	Reference	–	–
Luminal B	0.954	0.579–1.570	0.852	1.450	0.554–3.790	0.449
HER2+	2.019	1.388–2.936	<0.001	2.593	1.106–6.075	0.028
TNBC	1.903	1.222–2.964	0.004	3.094	1.536–6.232	0.002
Clinical stage						
Stage I–II	Reference	–	–	Reference	–	–
Stage III–IV	1.607	1.019–2.534	0.041	0.863	0.412–1.807	0.695
T stage						
T1	Reference	–	–	Reference	–	–
T2	1.294	0.812–2.064	0.279	1.185	0.678–2.072	0.552
T3	2.203	1.047–4.636	0.037	1.674	0.682–4.108	0.261
T4	1.123	0.389–3.244	0.830	4.084	0.723–23.056	0.111
N stage						
N0	Reference	–	–	Reference	–	–
N1–N3	1.822	1.127–2.947	0.014	1.875	0.999–3.520	0.048
M stage						
M0	Reference	–	–	–	–	–
M1	1.261	0.309–5.142	0.747	–	–	–
Chemotherapy						
Yes	0.681	0.428–1.082	0.104	0.424	0.164–1.091	0.075
No	Reference	–	–	Reference	–	–
Neoadjuvant chemotherapy						
Yes	1.392	0.954–2.031	0.087	1.002	0.420–2.391	0.997
No	Reference	–	–	Reference	–	–
Endocrine therapy						
Yes	0.554	0.404–0.759	<0.001	1.195	0.680–2.100	0.536
No	Reference	–	–	Reference	–	–
Targeted therapy						
Yes	1.367	0.976–1.913	0.069	0.781	0.378–1.613	0.504
No	Reference	–	–	Reference	–	–
Breast radiotherapy						
Yes	0.988	0.728–1.341	0.937	–	–	–
No	Reference	–	–	–	–	–
Breast surgery						
Mastectomy	0.697	0.437–1.112	0.130	0.350	0.132–0.929	0.035
Lumpectomy	0.913	0.492–1.693	0.772	0.432	0.140–1.333	0.144
No	Reference	–	–	Reference	–	–

## 4 DISCUSSION

This retrospective single‐center analysis examined various primary cancer characteristics, including age, sex, pathologic subtype, molecular features, clinical stage, TNM stage, and therapeutic methods, that are associated with interval time to the occurrence of BM. This was a large‐scale study that focused on TPDBM in all LC and BC subtypes at the same time. Moreover, compared with previous studies, which mainly discussed the impact of pathologic subtype on TPDBM, we also incorporated molecular alterations and detailed treatment modalities into multivariate analysis. Over the past decade, the improvement of comprehensive therapies has prolonged the overall survival of patients with LC and BC. This leads to the high incidence of BM, a common cause of morbidity and mortality. We believe that the present study may help to identify patients who have early development of BM. Early detection and intervention of these high‐risk patients are critical and beneficial.

Our results demonstrate that LC has an obviously shorter TPDBM than BC (median 5.3 months vs. 44.0 months, *p* < 0.001). For LC patients, median TPDBM is longest in SCLC (7.5 months), followed by LUSC (5.5 months), other NSCLC subtypes (5.0 months), and LUAD (3.0 months). However, in all LC patients, the pathologic subtype was not an independent predictor for TPDBM in our results. Another study suggested that nonadenocarcinomatous histopathology was a risk factor for the earlier development of BM in NSCLC.^16^ As previously reported, we also found that the pathologic subtype is an important factor that impacts the development of BCBM. The median TPDBM for luminal A, luminal B, HER2+, and TNBC was 56, 68, 39, and 33.5 months, respectively. In other studies, TNBC also has a short TPDBM, ranging from 14 to 35 months.^15^, ^22^, ^23^ Cao et al.^22^ revealed that TNBC (HR = 3.062, *p* < 0.001) and HER2+ (HR = 2.639, *p* = 0.009) were associated with early development of BC. In our multivariate analysis, TNBC (HR = 3.094, *p* = 0.002) and HER2+ (HR = 2.593, *p* = 0.028) were still significant risk factors. Studies also indicate that the incidence of BM varies in different primary tumor subtypes. TNBC is more likely to develop BM than the other types^24^ and EGFR mutation NSCLC has a higher incidence of BM than EGFR wild‐type NSCLC.^25^ Our research focused on the time course of BM progression, and LC and BC patients without BM were excluded. Therefore, we did not compare the variance in BM incidence in different cancer subtypes.

In addition, we investigated the impact of various key molecules on the interval time to BM. The molecular targeted approach has substantially changed the standards of care treatment of LC in recent years.^26^, ^27^ Accumulating evidence has shown its effectiveness in improving progression‐free survival and overall survival.^28^ EGFR mutations, KRAS mutations, and ALK rearrangements are the most frequent molecular alterations in NSCLC, particularly in LUAD. EGFR and KRAS mutations in primary LC are associated with prognosis.^29^ However, we detected that the mutation status of EGFR, KRAS, and ALK had no significant influence on TPDBM (Table S1). These findings are consistent with Smith's research.^16^ However, a multi‐institutional analysis detected a significant correlation between both EGFR and ALK gene alterations and interval time to subsequent BM.^30^ In our BCBM patient data, we examined the expression of an important proliferation marker, Ki67, in which ≥0.3 was regarded as a high expression. A high Ki67 index is associated with poor BC prognosis, regardless of the timing of specimen examination (i.e., pre/postoperative examination).^31^, ^32^ However, whether Ki67 can act as an independent predictive factor in the development of BM remains unclear. Our proportional hazards model indicates that a high level of Ki67 in primary BC correlates strongly with early occurrence of BM (HR = 3.024, *p* < 0.001), suggesting that a Ki67 index of more than 0.3 could serve as an independent risk factor to predict the development of BM.

Novel treatments for cancer patients, especially targeted therapy and immunotherapy, have developed quickly in recent years.^33^, ^34^ One advantage of our study is that all selected patients were diagnosed with BM between 2016 and 2020 and treated according to generally accepted management. Therefore, our findings could better reflect the contemporary BCBM and LCBM patient population undergoing current therapeutic procedures than previous studies. This may explain why patients in our cohort had a longer TPDBM than previous studies, in which patients were recruited before 2013.^14^, ^35^ Novel systemic therapies targeting primary lesions generally improve overall survival and progression‐free survival.^1^, ^36^ The majority of published studies focused solely on NSCLC. They revealed that advanced clinical stage, higher T and N stage, and larger primary tumor volume are potential predictors of TPDBM,^37^, ^38^, ^39^ which are similar to our results. However, limited information is known about the impact of different treatment approaches on the interval time to BM. Only a few studies suggest that induction chemotherapy is associated with the development of BM.^16^, ^38^ We first demonstrate that therapeutic approaches targeting primary LC, including surgery (*p* = 0.002), chemotherapy after surgery (*p* = 0.038), chemotherapy prior to and after surgery (*p* < 0.001), radiotherapy (*p* = 0.002), and targeted agents (*p* < 0.001), delay the occurrence of BM in all LC subtypes. However, among all treatment methods, BC patients could only benefit from mastectomy in terms of TPDBM (*p* = 0.035).

Several studies have demonstrated the prognostic factors associated with a high risk of BM. In LC, patients with EGFR mutation, larger primary lung lesions, and higher CEA concentration were more likely to have BM.^7^, ^9^ Young age, ER−, HER2+, tumor size >5 cm, and higher presenting stage are regarded as independent risk factors for BCBM.^8^, ^40^, ^41^ Our research is a good complementary for studying the progression of BM. Previous studies can help us to screen out the susceptible population for BM and we further identify the patients who may suffer from early BM. For these high‐risk patients, additional adjuvant treatments, early intervention, and more intensive follow‐up are needed to prevent BM progression in advance. The results of the present study could provide some novel references for the clinical management of BM.

In light of our findings that certain clinical, molecular, and therapeutic factors significantly influence the TPDBM, it is imperative to consider prophylactic treatment strategies for high‐risk LC and BC patients. Early intervention may prevent or delay the development of BMs, potentially improving patient outcomes. Based on the data, patients with high‐risk factors for early BMs should be considered for proactive surveillance and potentially preventative therapy. Additionally, the use of targeted therapies, which have shown efficacy in delaying the TPDBM, should be integrated into the treatment regimen for eligible patients. Furthermore, the implementation of regular MRI scans could aid in the early detection of BMs, allowing for timely intervention. Overall, integrating prophylactic strategies into the standard care protocol requires careful consideration of the patient's overall health, cancer subtype, and risk profile. Collaborative efforts between oncologists, radiologists, and neurologists are essential to tailor the most effective prophylactic treatment plans for these high‐risk patient populations.

There are some deficiencies in the present study. First, this is a single‐institution analysis, which limits the generalizability and applicability of the findings. More research with a larger number of cases from multiple centers is needed to confirm our results. Additionally, survival time data are not completed, and therefore not analyzed. In the future, we will investigate predictive factors for BM patient survival.

## 5 CONCLUSIONS

With the increase in incidence, BM is an intractable medical problem. The present study identifies the risk factors for early BM development in patients with LC and BC. For these high‐risk patients, early interventions are warranted and may lead to clinical benefits.

## AUTHOR CONTRIBUTIONS

**Haitao Ge:** Formal analysis (equal); funding acquisition (lead); methodology (equal); project administration (equal); writing – original draft (equal). **Kaibin Zhu:** Formal analysis (equal); validation (equal). **Qian Sun:** Data curation (equal); validation (equal). **Huan Wang:** Investigation (equal); validation (equal). **Hui Liu:** Formal analysis (equal); investigation (equal); validation (equal). **Jinyi Ge:** Investigation (equal); validation (equal). **Chunyang Liu:** Formal analysis (equal); validation (equal). **Peng Liang:** Conceptualization (equal); project administration (equal); resources (equal); writing – review and editing (equal). **Zhonghua Lv:** Conceptualization (equal); data curation (equal); funding acquisition (lead); resources (equal); supervision (equal); writing – review and editing (equal). **Hongbo Bao:** Conceptualization (lead); data curation (lead); formal analysis (lead); methodology (equal); resources (equal); visualization (lead); writing – review and editing (lead).

## FUNDING INFORMATION

Haitao Ge was supported by China Postdoctoral Science Foundation (2018M641845) and Postdoctoral Science Foundation of Heilongjiang Province (LBH‐Z18135). Zhonghua Lv was supported by Haiyan Foundation of Harbin Medical University Cancer Hospital (JJMS2021‐29) and Science and Technology Plan of Heilongjiang Provincial Health Commission (20230404040241).

## CONFLICT OF INTEREST STATEMENT

The authors have no relevant financial or non‐financial interests to disclose.

## ETHICS APPROVAL

This study was performed in line with the principles of the Declaration of Helsinki. Approval was granted by the Ethics Committee of Harbin Medical University Cancer Hospital (KY2021‐42).

## PATIENT CONSENT STATEMENT

Informed consent was obtained from all individual participants included in the study.

## Supporting information


**Table S1.** Univariate and multivariate analysis of TPDBM in LUAD.

## Data Availability

Data analyzed during the current study are available from the corresponding author on reasonable request.

## References

[1] Soerjomataram I , Bray F . Planning for tomorrow: global cancer incidence and the role of prevention 2020–2070. Nat Rev Clin Oncol. 2021;18(10):663‐672. 34079102 10.1038/s41571-021-00514-z

[2] Lah TT , Novak M , Breznik B . Brain malignancies: glioblastoma and brain metastases. Semin Cancer Biol. 2020;60:262‐273. 31654711 10.1016/j.semcancer.2019.10.010

[3] Stella GM , Corino A , Berzero G , Kolling S , Filippi AR , Benvenuti S . Brain metastases from lung cancer: is MET an actionable target? Cancer. 2019;11(3):271‐288. 10.3390/cancers11030271 PMC6468667 30813513

[4] Hines SL , Vallow LA , Tan WW , McNeil RB , Perez EA , Jain A . Clinical outcomes after a diagnosis of brain metastases in patients with estrogen‐ and/or human epidermal growth factor receptor 2‐positive versus triple‐negative breast cancer. Ann Oncol. 2008;19(9):1561‐1565. 18534964 10.1093/annonc/mdn283

[5] Fares J , Ulasov I , Timashev P , Lesniak MS . Emerging principles of brain immunology and immune checkpoint blockade in brain metastases. Brain. 2021;144(4):1046‐1066. 33893488 10.1093/brain/awab012 PMC8105040

[6] Lamba N , Wen PY , Aizer AA . Epidemiology of brain metastases and leptomeningeal disease. Neuro‐Oncology. 2021;23(9):1447‐1456. 33908612 10.1093/neuonc/noab101 PMC8408881

[7] Ge M , Zhuang Y , Zhou X , Huang R , Liang X , Zhan Q . High probability and frequency of EGFR mutations in non‐small cell lung cancer with brain metastases. J Neuro‐Oncol. 2017;135(2):413‐418. 10.1007/s11060-017-2590-x 28780743

[8] Soffietti R , Ahluwalia M , Lin N , Ruda R . Management of brain metastases according to molecular subtypes. Nat Rev Neurol. 2020;16(10):557‐574. 32873927 10.1038/s41582-020-0391-x

[9] He J , Wang X , Xiao R , Zuo W , Zhang W , Yao H . Risk factors for brain metastases from non‐small‐cell lung cancer: a protocol for observational study. Medicine (Baltimore). 2021;100(9):e24724. 33655937 10.1097/MD.0000000000024724 PMC7939174

[10] Li M , Song Y , Li L , Qin J , Deng H , Zhang T . Reirradiation of whole brain for recurrent brain metastases: a case report of lung cancer with 12‐year survival. Front Oncol. 2021;11:780581. 34900735 10.3389/fonc.2021.780581 PMC8660684

[11] McKay MJ . Brain metastases: increasingly precision medicine‐a narrative review. Ann Transl Med. 2021;9(21):1629. 34926673 10.21037/atm-21-3665 PMC8640905

[12] Narkhede AA , Crenshaw JH , Crossman DK , Shevde LA , Rao SS . An in vitro hyaluronic acid hydrogel based platform to model dormancy in brain metastatic breast cancer cells. Acta Biomater. 2020;107:65‐77. 32119920 10.1016/j.actbio.2020.02.039

[13] Tsuzuki N , Miyazawa T , Nawashiro H , Shima K . A metastatic dormant tumour in the brain. Lancet Oncol. 2000;1:147. 11905652 10.1016/s1470-2045(00)00036-x

[14] Ahn HK , Park YH , Lee SJ , et al. Clinical implication of time to brain metastasis (TTBM) according to breast cancer subtypes. Springerplus. 2013;2(1):136. 23667803 10.1186/2193-1801-2-136 PMC3647103

[15] Berghoff A , Bago‐Horvath Z , De Vries C , et al. Brain metastases free survival differs between breast cancer subtypes. Br J Cancer. 2012;106(3):440‐446. 22233926 10.1038/bjc.2011.597 PMC3273356

[16] Smith DR , Bian Y , Wu CC , et al. Natural history, clinical course and predictors of interval time from initial diagnosis to development of subsequent NSCLC brain metastases. J Neuro‐Oncol. 2019;143(1):145‐155. 10.1007/s11060-019-03149-4 30874953

[17] Fecci PE , Champion CD , Hoj J , et al. The evolving modern management of brain metastasis. Clin Cancer Res. 2019;25(22):6570‐6580. 31213459 10.1158/1078-0432.CCR-18-1624 PMC8258430

[18] Nicholson AG , Tsao MS , Beasley MB , et al. The 2021 WHO classification of lung tumors: impact of advances since 2015. J Thorac Oncol. 2022;17(3):362‐387. 34808341 10.1016/j.jtho.2021.11.003

[19] Harbeck N , Penault‐Llorca F , Cortes J , et al. Breast cancer. Nat Rev Dis Primers. 2019;5(1):66. 31548545 10.1038/s41572-019-0111-2

[20] Waks AG , Winer EP . Breast cancer treatment: a review. JAMA. 2019;321(3):288‐300. 30667505 10.1001/jama.2018.19323

[21] Giuliano AE , Edge SB , Hortobagyi GN . Eighth edition of the AJCC cancer staging manual: breast cancer. Ann Surg Oncol. 2018;25(7):1783‐1785. 29671136 10.1245/s10434-018-6486-6

[22] Cao TQ , Dixit K , Santa‐Maria C , Kumthekar P . Factors affecting time to brain metastases for stage 2 and 3 breast cancer patients: a large single‐institutional analysis with potential screening implications. Neurooncol Adv. 2021;3(1):vdab009. 33738445 10.1093/noajnl/vdab009 PMC7954098

[23] Hulsbergen AFC , Lamba N , Claes A , et al. Prognostic value of brain metastasis‐free interval in patients with breast cancer brain metastases. World Neurosurg. 2019;128:e157‐e164. 31035019 10.1016/j.wneu.2019.04.072

[24] Martin AM , Cagney DN , Catalano PJ , et al. Brain metastases in newly diagnosed breast cancer: a population‐based study. JAMA Oncol. 2017;3(8):1069‐1077. 28301662 10.1001/jamaoncol.2017.0001 PMC5824221

[25] Iuchi T , Shingyoji M , Itakura M , et al. Frequency of brain metastases in non‐small‐cell lung cancer, and their association with epidermal growth factor receptor mutations. Int J Clin Oncol. 2015;20(4):674‐679. 25336382 10.1007/s10147-014-0760-9

[26] Tan AC , Tan DSW . Targeted therapies for lung cancer patients with oncogenic driver molecular alterations. J Clin Oncol. 2022;40(6):611‐625. 34985916 10.1200/JCO.21.01626

[27] Hirsch FR , Suda K , Wiens J , Bunn PA Jr . New and emerging targeted treatments in advanced non‐small‐cell lung cancer. Lancet. 2016;388(10048):1012‐1024. 27598681 10.1016/S0140-6736(16)31473-8

[28] Hanna NH , Robinson AG , Temin S , et al. Therapy for stage IV non‐small‐cell lung cancer with driver alterations: ASCO and OH (CCO) joint guideline update. J Clin Oncol. 2021;39(9):1040‐1091. 33591844 10.1200/JCO.20.03570

[29] Sperduto PW , Yang TJ , Beal K , et al. Estimating survival in patients with lung cancer and brain metastases: an update of the graded prognostic assessment for lung cancer using molecular markers (lung‐molGPA). JAMA Oncol. 2017;3(6):827‐831. 27892978 10.1001/jamaoncol.2016.3834 PMC5824323

[30] Sperduto PW , Yang TJ , Beal K , et al. The effect of gene alterations and tyrosine kinase inhibition on survival and cause of death in patients with adenocarcinoma of the lung and brain metastases. Int J Radiat Oncol Biol Phys. 2016;96(2):406‐413. 27598807 10.1016/j.ijrobp.2016.06.006 PMC5575932

[31] Choi SB , Park JM , Ahn JH , et al. Ki‐67 and breast cancer prognosis: does it matter if Ki‐67 level is examined using preoperative biopsy or postoperative specimen? Breast Cancer Res Treat. 2022;192:343‐352. 35025005 10.1007/s10549-022-06519-1 PMC8926964

[32] Erices‐Leclercq M , Lubig S , Forster F , et al. Prognostic relevance of Ki67 expression in primary male breast cancer: determination of cut‐off points by different evaluation methods and statistical examinations. J Cancer Res Clin Oncol. 2022;148(2):441‐447. 33991247 10.1007/s00432-021-03623-5 PMC11800765

[33] Piccart‐Gebhart MJ , Procter M , Leyland‐Jones B , et al. Trastuzumab after adjuvant chemotherapy in HER2‐positive breast cancer. N Engl J Med. 2005;353(16):1659‐1672. 16236737 10.1056/NEJMoa052306

[34] Chaft JE , Shyr Y , Sepesi B , Forde PM . Preoperative and postoperative systemic therapy for operable non‐small‐cell lung cancer. J Clin Oncol. 2022;40(6):546‐555. 34985966 10.1200/JCO.21.01589 PMC8853628

[35] Sperduto PW , Kased N , Roberge D , et al. The effect of tumor subtype on the time from primary diagnosis to development of brain metastases and survival in patients with breast cancer. J Neuro‐Oncol. 2013;112(3):467‐472. 10.1007/s11060-013-1083-9 23462853

[36] Suh JH , Kotecha R , Chao ST , Ahluwalia MS , Sahgal A , Chang EL . Current approaches to the management of brain metastases. Nat Rev Clin Oncol. 2020;17(5):279‐299. 32080373 10.1038/s41571-019-0320-3

[37] Kim M , Suh CH , Lee SM , et al. Development of brain metastases in patients with non‐small cell lung cancer and no brain metastases at initial staging evaluation: cumulative incidence and risk factor analysis. AJR Am J Roentgenol. 2021;217(5):1184‐1193. 34037408 10.2214/AJR.21.25787

[38] Ji Z , Bi N , Wang J , et al. Risk factors for brain metastases in locally advanced non‐small cell lung cancer with definitive chest radiation. Int J Radiat Oncol Biol Phys. 2014;89(2):330‐337. 24725335 10.1016/j.ijrobp.2014.02.025

[39] Won YW , Joo J , Yun T , et al. A nomogram to predict brain metastasis as the first relapse in curatively resected non‐small cell lung cancer patients. Lung Cancer. 2015;88(2):201‐207. 25726044 10.1016/j.lungcan.2015.02.006

[40] Koniali L , Hadjisavvas A , Constantinidou A , et al. Risk factors for breast cancer brain metastases: a systematic review. Oncotarget. 2020;11(6):650‐669. 32110283 10.18632/oncotarget.27453 PMC7021234

[41] Maurer C , Tulpin L , Moreau M , et al. Risk factors for the development of brain metastases in patients with HER2‐positive breast cancer. ESMO Open. 2018;3(6):e000440. 30425844 10.1136/esmoopen-2018-000440 PMC6212674

