# Supplementary material for: The clinical, molecular, and therapeutic implications of time from primary diagnosis to brain metastasis in lung and breast cancer patients
Source: Cancer Med. 2024 Jun 7;13(11):e7364. doi: 10.1002/cam4.7364 (PMC11157198; doi:10.1002/cam4.7364)
Supplement: Supplementary file 1 — Table S1. Univariate and multivariate analysis of TPDBM in LUAD. [file CAM4-13-e7364-s001.docx]

**Supplementary Table 1. Univariate and multivariate analysis of TPDBM in LUAD**

|  | **Univariate analysis** | | |  | **Multivariate analysis** | | |
| --- | --- | --- | --- | --- | --- | --- | --- |
|  | **HR** | **95% CI** | **P-value** |  | **HR** | **95% CI** | **P-value** |
| **Age (y)** |  |  |  |  |  |  |  |
| <60 | Reference | - | - |  | - | - | - |
| ≥60 | 0.952 | 0.766-1.183 | 0.194 |  | - | - | - |
| **Gender** |  |  |  |  |  |  |  |
| Female | Reference | - | - |  | - | - | - |
| Male | 1.035 | 0.836-1.282 | 0.751 |  | - | - | - |
| **Clinical stage** |  |  |  |  |  |  |  |
| Stage I | Reference | - | - |  | Reference | - | - |
| Stage II | 1.214 | 0.420-3.511 | 0.721 |  | 0.851 | 0.122-5.962 | 0.871 |
| Stage III | 1.377 | 0.579-3.274 | 0.469 |  | 1.002 | 0.224-4.482 | 0.998 |
| Stage IV | 2.858 | 1.265-6.457 | 0.012 |  | 1.661 | 0.359-7.694 | 0.516 |
| **T stage** |  |  |  |  |  |  |  |
| 1 | Reference | - | - |  | Reference | - | - |
| 2 | 1.361 | 1.038-1.784 | 0.026 |  | 1.517 | 1.038-2.216 | 0.031 |
| 3 | 0.966 | 0.600-1.555 | 0.886 |  | 1.662 | 0.857-3.224 | 0.133 |
| 4 | 0.939 | 0.623-1.416 | 0.764 |  | 1.082 | 0.621-1.883 | 0.781 |
| **N stage** |  |  |  |  |  |  |  |
| 0 | Reference | - | - |  | Reference | - | - |
| 1 | 0.553 | 0.298-1.025 | 0.060 |  | 0.994 | 0.363-2.723 | 0.991 |
| 2 | 1.281 | 0.914-1.795 | 0.150 |  | 1.293 | 0.771-2.166 | 0.330 |
| 3 | 1.491 | 1.039-2.140 | 0.030 |  | 1.492 | 0.888-2.509 | 0.131 |
| **M stage** |  |  |  |  |  |  |  |
| 0 | Reference | - | - |  | Reference | - | - |
| 1 | 2.303 | 1.683-3.150 | <0.001 |  | 1.661 | 0.359-7.694 | 0.516 |
| **Chemotherapy** |  |  |  |  |  |  |  |
| After surgery | 0.255 | 0.163-0.399 | <0.001 |  | 0.714 | 0.229-2.224 | 0.561 |
| Before and after surgery | 0.714 | 0.547-0.931 | 0.013 |  | 0.576 | 0.360-0.920 | 0.021 |
| None | Reference | - | - |  | Reference | - | - |
| **Targeted therapy** |  |  |  |  |  |  |  |
| Yes | 0.698 | 0.555-0.856 | 0.001 |  | 0.648 | 0.435-0.964 | 0.033 |
| No | Reference | - | - |  | Reference | - | - |
| **Pulmonary radiotherapy** |  |  |  |  |  |  |  |
| Yes | 0.867 | 0.685-1.097 | 0.236 |  | 0.647 | 0.443-0.943 | 0.024 |
| No | Reference | - | - |  | Reference | - | - |
| **Pulmonary surgery** |  |  |  |  |  |  |  |
| Yes | 0.344 | 0.244-0.484 | <0.001 |  | 0.391 | 0.126-1.219 | 0.106 |
| No | Reference | - | - |  | Reference | - | - |
| **EGFR status** |  |  |  |  |  |  |  |
| Wild type | Reference | - | - |  | Reference | - | - |
| Mutation | 0.617 | 0.462-0.824 | 0.001 |  | 0.811 | 0.543-1.213 | 0.309 |
| **KRAS status** |  |  |  |  |  |  |  |
| Wild type | Reference | - | - |  | - | - | - |
| Mutation | 0.522 | 0.269-1.016 | 0.056 |  | - | - | - |
| **ALK status** |  |  |  |  |  |  |  |
| Wild type | Reference | - | - |  | - | - | - |
| Rearrangements | 0.975 | 0.535-1.779 | 0.935 |  | - | - | - |
